# Supplementary material for: Life on the margin: Rainwater tanks facilitate overwintering of the dengue vector, Aedes aegypti, in a sub-tropical climate
Source: PLoS One. 2019 Apr 25;14(4):e0211167. doi: 10.1371/journal.pone.0211167 (PMC6483192; doi:10.1371/journal.pone.0211167)
Supplement: S1 Table — Environmental chamber temperatures used to determine survival of Aedes aegypti in different container categories from Brisbane, Australia. (DOCX) [file pone.0211167.s001.docx]

**S1. Table. Temperature Regimes.** Temperature regimes for environmental chambers to determine survival of *Aedes aegypti* in different container categories from Brisbane, Australia.
