# Supplementary material for: Life on the margin: Rainwater tanks facilitate overwintering of the dengue vector, Aedes aegypti, in a sub-tropical climate
Source: PLoS One. 2019 Apr 25;14(4):e0211167. doi: 10.1371/journal.pone.0211167 (PMC6483192; doi:10.1371/journal.pone.0211167)
Supplement: S2 Table — Abiotic conditions within rainwater tanks and buckets in Brisbane during winter (July) 2014. Levels represent 0–32% shade cover (1), 33–65% (2) and 66–100% (3). (DOCX) [file pone.0211167.s002.docx]

**S2. Table. Rainwater Tank Conditions.** Abiotic conditions within rainwater tanks and buckets in Brisbane during winter (July) 2014. Levels represent 0-32% shade cover (1), 33-65% (2) and 66-100% (3).
