# Supplementary material for: Life on the margin: Rainwater tanks facilitate overwintering of the dengue vector, Aedes aegypti, in a sub-tropical climate
Source: PLoS One. 2019 Apr 25;14(4):e0211167. doi: 10.1371/journal.pone.0211167 (PMC6483192; doi:10.1371/journal.pone.0211167)
Supplement: S3 Table — Temperatures recorded in air at Archerfield Airport, (-27.57o S, 153.01o E), tanks and buckets from Brisbane during winter (1st June until 31st August), 2014. (DOCX) [file pone.0211167.s003.docx]

**S3. Table. Weather Records.** Temperatures recorded in air at Archerfield Airport, (-27.57°S, 153.01° E), tanks and buckets from Brisbane and Adelaide Airport (-34.95° S, 138.52° E) during winter (1st June until 31st August), 2014.

| **Location** | **Mean °C (SD)** | **Maximum Daily Range (°C)** | **Mean Daily Maximum °C (SD)** | **Mean Daily Minimum °C (SD)** |
| --- | --- | --- | --- | --- |
| Brisbane Air | 15.6 (2.02) | 23.0 | 22.8 (2.1) | 8.4 (3.4) |
| Brisbane Tanks | 16.9 (1.89) | 11.3 | 19.7 (1.4) | 14.5 (1.7) |
| Brisbane Buckets | 16.2 (4.10) | 29.9 | 28.5 (3.4) | 10.7 (2.7) |
