# Supplementary material for: Life on the margin: Rainwater tanks facilitate overwintering of the dengue vector, Aedes aegypti, in a sub-tropical climate
Source: PLoS One. 2019 Apr 25;14(4):e0211167. doi: 10.1371/journal.pone.0211167 (PMC6483192; doi:10.1371/journal.pone.0211167)
Supplement: S4 Table — Shading represents presence during larval surveys conducted fortnightly. All tanks were sealed, and tanks 1 and 8 had inflows of water removed after the first survey. (DOCX) [file pone.0211167.s004.docx]

**S4. Table. Presence/absence of *Aedes notoscriptus* immatures in sealed rainwater tanks during winter in Brisbane, 2014**. Shading represents presence during larval surveys conducted fortnightly. All tanks were sealed, and tanks 1 and 8 had inflows of water removed after the first survey.

**

**
