# Supplementary material for: Life on the margin: Rainwater tanks facilitate overwintering of the dengue vector, Aedes aegypti, in a sub-tropical climate
Source: PLoS One. 2019 Apr 25;14(4):e0211167. doi: 10.1371/journal.pone.0211167 (PMC6483192; doi:10.1371/journal.pone.0211167)
Supplement: S5 Table — Presence of Aedes notoscriptus immatures (grey shading) in first flush devices during winter in Brisbane, 2014. Numbers correspond to the tank which contained devices. For example, tank 10 had two separate first flush devices (10a,10b) on downpipes entering tank. Volume measures the mean volume found in each device throughout the field survey. Presence represents the percentage of surveys where at least one Ae. notoscriptus immature was sampled from the device. (DOCX) [file pone.0211167.s005.docx]

**S5. Table. Mosquito Presence in Rainwater Tanks.** Presence of *Aedes notoscriptus* immatures (grey shading) in first flush devices during winter in Brisbane, 2014. Numbers correspond to the tank which contained devices. For example, tank 10 had two separate first flush devices (10a,10b) on downpipes entering tank. Volume measures the mean volume found in each device throughout the field survey. Presence represents the percentage of surveys where at least one *Ae. notoscriptus* immature was sampled from the device.
