# Supplementary material for: Life on the margin: Rainwater tanks facilitate overwintering of the dengue vector, Aedes aegypti, in a sub-tropical climate
Source: PLoS One. 2019 Apr 25;14(4):e0211167. doi: 10.1371/journal.pone.0211167 (PMC6483192; doi:10.1371/journal.pone.0211167)
Supplement: S6 Table — Survival of tropical and subtropical Aedes aegypti strains in rainwater tank (small fluctuation), buckets (large fluctuation) and 26°C control (constant) treatments. (DOCX) [file pone.0211167.s006.docx]

**S6. Table. *Aedes aegypti* Survival.** Survival of tropical and subtropical *Aedes aegypti* strains in rainwater tank (small fluctuation), buckets (large fluctuation) and 26°C control (constant) treatments.

| **Treatment** | **Strain/Species** | **Adults Surviving** | **Deaths** | **Total** | **%** |
| --- | --- | --- | --- | --- | --- |
| Tanks | Subtropical *Ae. aegypti* | 268 | 134 | 402 | 66.7 |
|  | Tropical *Ae. aegypti* | 291 | 94 | 385 | 75.6 |
| Bucket | Subtropical *Ae. aegypti* | 193 | 207 | 400 | 48.3 |
|  | Tropical *Ae. aegypti* | 274 | 141 | 415 | 66.0 |
| Control | Subtropical *Ae. aegypti* | 391 | 11 | 402 | 97.3 |
|  | Tropical *Ae. aegypti* | 389 | 4 | 393 | 99.0 |
