# Supplementary material for: Life on the margin: Rainwater tanks facilitate overwintering of the dengue vector, Aedes aegypti, in a sub-tropical climate
Source: PLoS One. 2019 Apr 25;14(4):e0211167. doi: 10.1371/journal.pone.0211167 (PMC6483192; doi:10.1371/journal.pone.0211167)
Supplement: S7 Table — Mean, standard error, minimum and maximum development time for tropical and subtropical Aedes aegypti strains in rainwater tank (small fluctuation), buckets (large fluctuation) and 26°C control (constant) treatments. (DOCX) [file pone.0211167.s007.docx]

**S7. Table. *Aedes aegypti* Development.** Mean, standard error, minimum and maximum development time for tropical and subtropical *Aedes aegypti* strains in rainwater tank (small fluctuation), buckets (large fluctuation) and 26°C control (constant) treatments.

| **Treatment** | **Strain/Species** | **Mean Days to Adult** | **SE** | **Min** | **Max** |
| --- | --- | --- | --- | --- | --- |
| Tanks | Subtropical *Ae. aegypti* | 32.48 | 0.19 | 26 | 46 |
|  | Tropical *Ae. aegypti* | 32.70 | 0.20 | 23 | 58 |
| Bucket | Subtropical *Ae. aegypti* | 32.22 | 0.23 | 27 | 54 |
|  | Tropical *Ae. aegypti* | 31.37 | 0.18 | 26 | 47 |
| Control | Subtropical *Ae. aegypti* | 8.88 | 0.03 | 7 | 11 |
|  | Tropical *Ae. aegypti* | 9.05 | 0.04 | 7 | 15 |
