# Supplementary material for: Life on the margin: Rainwater tanks facilitate overwintering of the dengue vector, Aedes aegypti, in a sub-tropical climate
Source: PLoS One. 2019 Apr 25;14(4):e0211167. doi: 10.1371/journal.pone.0211167 (PMC6483192; doi:10.1371/journal.pone.0211167)
Supplement: S1 Fig — Roof gutters observed that likely increased productivity of rainwater tanks during winter in Brisbane, 2014. (DOCX) [file pone.0211167.s008.docx]

**S8.** **Productive Infrastructure.** Roof gutters observed that likely increased productivity of rainwater tanks during winter in Brisbane, 2014.

**
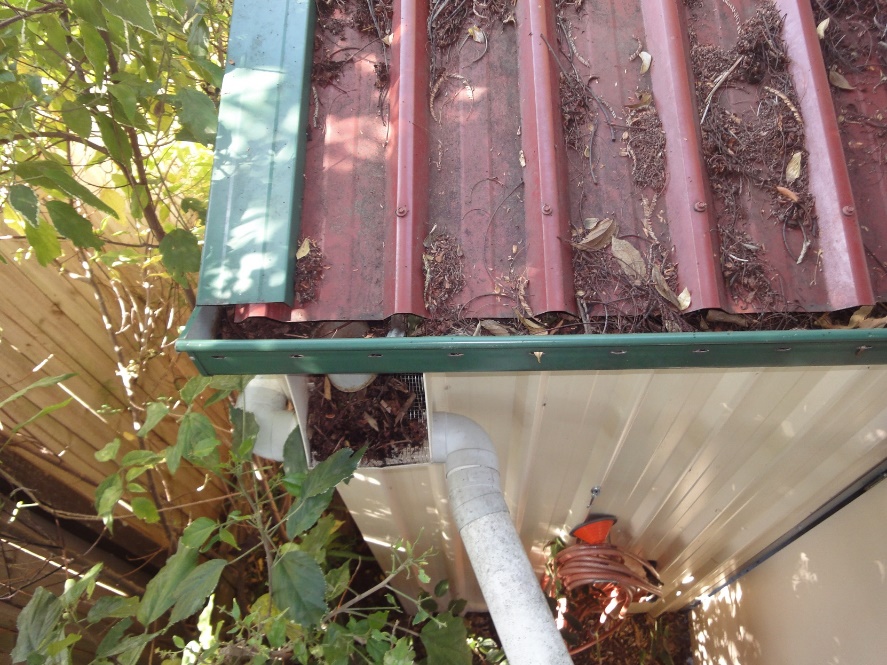

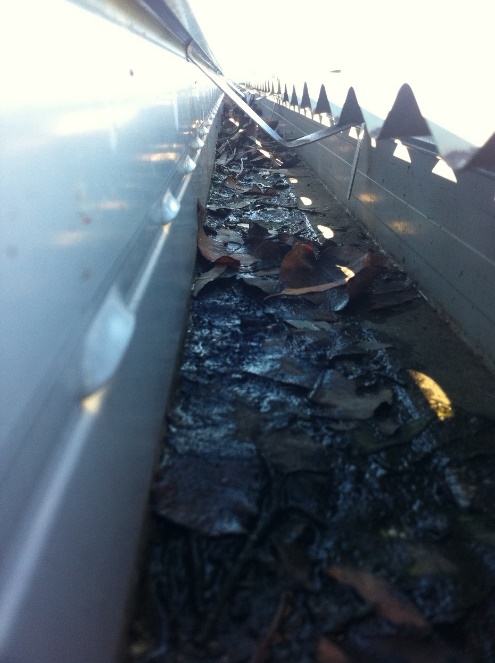
**
